# Supplementary material for: Planar Hall effect and anisotropic magnetoresistance in polar-polar interface of LaVO3-KTaO3 with strong spin-orbit coupling
Source: Nat Commun. 2020 Feb 13;11:874. doi: 10.1038/s41467-020-14689-z (PMC7018836; doi:10.1038/s41467-020-14689-z)
Supplement: Supplementary file 1 — Supplementary Information [file 41467_2020_14689_MOESM1_ESM.pdf]

Planar Hall Effect and Anisotropic Magnetoresistance in polar-polar interface of $LaVO_3$-$KTaO_3$ with strong spin-orbit coupling

Neha Wadehra,[1] Ruchi Tomar,[1] Rahul Mahavir Varma,[2] R. K. Gopal,[3] Yogesh Singh,[3] Sushanta Dattagupta,[4] and S. Chakraverty[1,*]

[1]*Nanoscale Physics and Device Laboratory, Institute of Nano Science and Technology, Phase-10, Sector-64, Mohali, Punjab, 160062, India.*

[2]*Solid State and Structural Chemistry Unit, Indian Institute of Science, Bangaluru, Karnataka, 560012, India.*

[3]*Indian Institute of Science Education and Research Mohali, Knowledge City, Sector-81, SAS Nagar, Manauli, 140306, India.*

[4]*Bose Institute, P-1/12, CIT Rd, Scheme VIIM, Kankurgachi, Kolkata, West Bengal-700054, India.*

(Dated: January 17, 2020)

*Electronic address: `suvankar.chakraverty@gmail.com`

Supplementary Information

Supplementary Note 1- For application of small magnetic field $\mathbf{B}_A$

The total Hamiltonian can be written as:

$$H = H_{\mathrm{o}} + V \tag{1}$$

where considering Supplementary Figure 1,

$$H_o = \epsilon(k) - \alpha(\sigma_{\mathrm{x}} k_{\mathrm{y}} - \sigma_{\mathrm{y}} k_{\mathrm{x}}) \tag{2}$$

$$\alpha = \mu_{\mathrm{B}} \hbar E / mc^2$$

$$V = -\mu_{\mathrm{B}} |\mathbf{B_A}| (\sigma_{\mathrm{x}} cos\phi - \sigma_{\mathrm{y}} sin\phi)) \tag{3}$$

H_{o} is the unperturbed Hamiltonian, V is the perturbation.

The eigenvalues of H_{o} are

$$\lambda_{\mathrm{o}}^{\pm} = \epsilon(k) \mp \alpha k = \hbar^2 k^2 / 2m \mp \alpha k \tag{4}$$

$$k = \sqrt{k_{\mathrm{x}}^2 + k_{\mathrm{y}}^2}$$

The corresponding eigenfunctions known as minority Rashba-band and majority Rashba-band are respectively given as:[1]

$$|k^+\rangle = \frac{1}{\sqrt{2}} \begin{pmatrix} 1 \\ -ie^{i\theta_k} \end{pmatrix} \, and \, |k^-\rangle = \frac{1}{\sqrt{2}} \begin{pmatrix} 1 \\ ie^{i\theta_k} \end{pmatrix} \tag{5}$$

Here,

$$cos\theta_k = \frac{k_{\mathrm{x}}}{k} \; and \; sin\theta_k = \frac{k_{\mathrm{y}}}{k} \tag{6}$$

Now refering to Taskin et al. supplementary notes[2], when a resistivity anisotropy is induced by an in-plane magnetic field $\mathbf{B_A}$ along **x'**-axis (Fig. 2), the resistivity tensor may be written in a diagonalized form:

$$\begin{pmatrix} E_{\mathrm{x'}} \\ E_{\mathrm{y'}} \end{pmatrix} = \begin{pmatrix} R_{\parallel} & 0 \\ 0 & R_{\perp} \end{pmatrix} \begin{pmatrix} j_{\mathrm{x'}} \\ j_{\mathrm{y'}} \end{pmatrix} \tag{7}$$

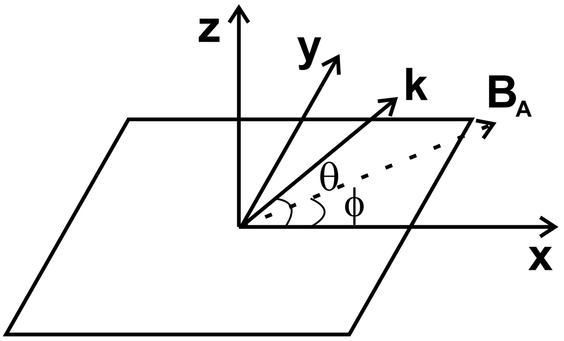

Supplementary Figure 1: **Schematic.** Schematic for the direction of propagation of carriers and applied magnetic field.

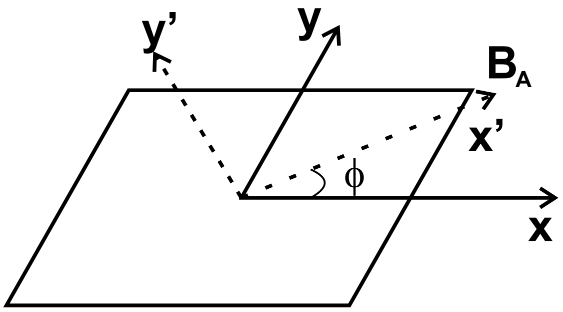

Supplementary Figure 2: **Schematic.** Schematic presenting the laboratory frame of reference and the frame of reference of the applied magnetic field.

Here, $E_{\mathrm{x'}}$ and $j_{\mathrm{x'}}$ are along $\mathbf{B_A}$ while $E_{\mathrm{y'}}$ and $j_{\mathrm{y'}}$ are perpendicular. Here, unlike Taskin et al., we provide explicit expressions for $R_{\|}$ and $R_{\perp}$ with reference to the Hamiltonian in Supplementary Eq.1-3.

Computation of $\boldsymbol{R}_{\perp}$ This part is the simplest because in this geometry, the electric field $\mathbf{E}$ is applied along **y'**-axis ($E_{\mathrm{y'}}$) and the current j is also measured in this direction ($j_{\mathrm{y'}}$). Hence, the magnetic field, being normal to **y'**-axis, has no influence on $R_{\perp}$, thereby making the Rashba physics irrelevant. Then we will take $R_{\perp}$ to be the simple Drude contribution R_o, which we do not calculate. So,

$$R_{\perp} = R_{\mathrm{o}} \tag{8}$$

Computation of $\boldsymbol{R}_{\|}$ Now, both the electric field $E_{\mathrm{x'}}$ and the current $j_{\mathrm{x'}}$ are along the direction in which the in-plane $\mathbf{B_A}$ field is applied. Therefore, in addition to the residual Drude resistivity R_{o}, the strong presence of the spin-orbit interaction via the Rashba effect comes into play.

Because $\mathbf{B_A}$ couples to σ_x' via the Zeeman interaction (Supplementary Eq. 3), it causes transitions between the majority and minority Rashba bands which are the eigenstates of the unperturbed Hamiltonian H_o (Supplementary Eq. 5). These transitions are like 'spin-flip' scattering processes which, in turn, cause momentum reversal (from **k** to **-k**) thereby causing resistance. In first order perturbation theory,

$$R_{\|} = R_o + \gamma\mu_B^2\ |\mathbf{B_A}|^2\ \overline{|\langle \underline{k}'^{-}|\,\sigma_x\,|\underline{k}'^{+}\rangle|^2} \tag{9}$$

γ being a constant, where from Eq. 5,

$$|\underline{k}'^{+}\rangle = \frac{1}{\sqrt{2}}\begin{pmatrix}1\\ -ie^{i\theta_{k'}}\end{pmatrix}\ and\,|\underline{k}'^{-}\rangle = \frac{1}{\sqrt{2}}\begin{pmatrix}1\\ ie^{i\theta_k'}\end{pmatrix} \tag{10}$$

In Supplementary Eq. 9, the Drude contribution R_o is added. The bar denotes an average over all k'-directions. Now,

$$\langle \underline{k}'^{-}|\,\sigma_x\,|\underline{k}'^{+}\rangle = \frac{1}{2}\begin{pmatrix}1 & -ie^{-i\theta_{k'}}\end{pmatrix}\begin{pmatrix}0 & 1\\ 1 & 0\end{pmatrix}\begin{pmatrix}1\\ -ie^{i\theta_{k'}}\end{pmatrix}$$
$$= \frac{1}{2}\begin{pmatrix}1 & -ie^{-i\theta_{k'}}\end{pmatrix}\begin{pmatrix}-ie^{i\theta_{k'}}\\ 1\end{pmatrix} = -icos\theta_k'$$

Therefore,

$$\overline{|\langle \underline{k}'^{-}|\,\sigma_x\,|\underline{k}'^{+}\rangle|^2} = \overline{cos^2\theta_{k'}} = \frac{1}{2} \tag{11}$$

Substituting,

$$R_{\|} = R_o + \frac{\gamma}{2}\mu_B^2\ B_A^2 \tag{12}$$

Following Taskin et al., the transformation from the principal axes to the film (i.e. laboratory axes) is effected by

$$\begin{pmatrix}E_x\\ E_y\end{pmatrix} = \begin{pmatrix}cos\phi & -sin\phi\\ sin\phi & cos\phi\end{pmatrix}\begin{pmatrix}R_{\|} & 0\\ 0 & R_{\perp}\end{pmatrix}\begin{pmatrix}cos\phi & sin\phi\\ -sin\phi & cos\phi\end{pmatrix}\begin{pmatrix}j_x\\ j_y\end{pmatrix} \tag{13}$$

By setting j_y=0, in confirmation with our experimental results, we get,

$$R_{xx} = \frac{E_x}{j_x} = R_{\perp} + (R_{\|} - R_{\perp})cos^2\phi \tag{14}$$

$$R_{yx} = \frac{E_y}{j_x} = (R_{\|} - R_{\perp})cos\phi sin\phi \tag{15}$$

From Eq. 12 and 8,

$$R_{\rm xx} = R_{\rm o} + \frac{\gamma}{2}\mu_{\rm B}^2\ B_{\rm A}^2\ cos^2\phi \tag{16}$$

$$R_{\rm yx} = \frac{\gamma}{2}\mu_{\rm B}^2\ cos\phi sin\phi \tag{17}$$

In Supplementary Eq. 14, R_o is expected to be small.

Supplementary Note 2- Calculation of transition matrices

The matrix elements for the transitions between the Rashba-energy split bands (1,2,3 and 4) shown in Figure 4(e) of manuscript are given by:

$$\langle 1|\,\sigma_{\rm y}\,|2\rangle = f_-^*(k) - f_-(k-2\Delta) \tag{18}$$

$$\langle 1|\,\sigma_{\rm y}\,|3\rangle = f_-^*(k) - f_+(k-2\Delta) \tag{19}$$

$$\langle 1|\,\sigma_{\rm y}\,|4\rangle = f_-^*(k) + f_+(k) \tag{20}$$

$$\langle 2|\,\sigma_{\rm y}\,|3\rangle = -f_-^*(k-2\Delta) - f_+(k-2\Delta) \tag{21}$$

$$\langle 2|\,\sigma_{\rm y}\,|4\rangle = -f_-^*(k-2\Delta) + f_+(k) \tag{22}$$

$$\langle 3|\,\sigma_{\rm y}\,|4\rangle = -f_+^*(k-2\Delta) + f_+(k) \tag{23}$$

where,

$$f_{(+,-)}(k) = \frac{1}{2}[\frac{k+(-)rsin(\Phi)-(+)ircos(\Phi)}{\sqrt{r^2+k^2+(-)2rksin(\Phi)}}] \tag{24}$$

and f* is its complex conjugate, **k** is wave vector, Δ is the split in the Rashba-energy bands, ϕ is the angle between applied current (I) and magnetic field (**B**) and $\sigma_{\rm y}$ is the Pauli-spin matrix.

Supplementary Note 3- X-ray photoelectron spectroscopy measurements

We have performed X-ray photoelectron spectroscopy (XPS) measurement on the freshly prepared LVO thin film. XPS measurement of the core level spectrum were carried out with a

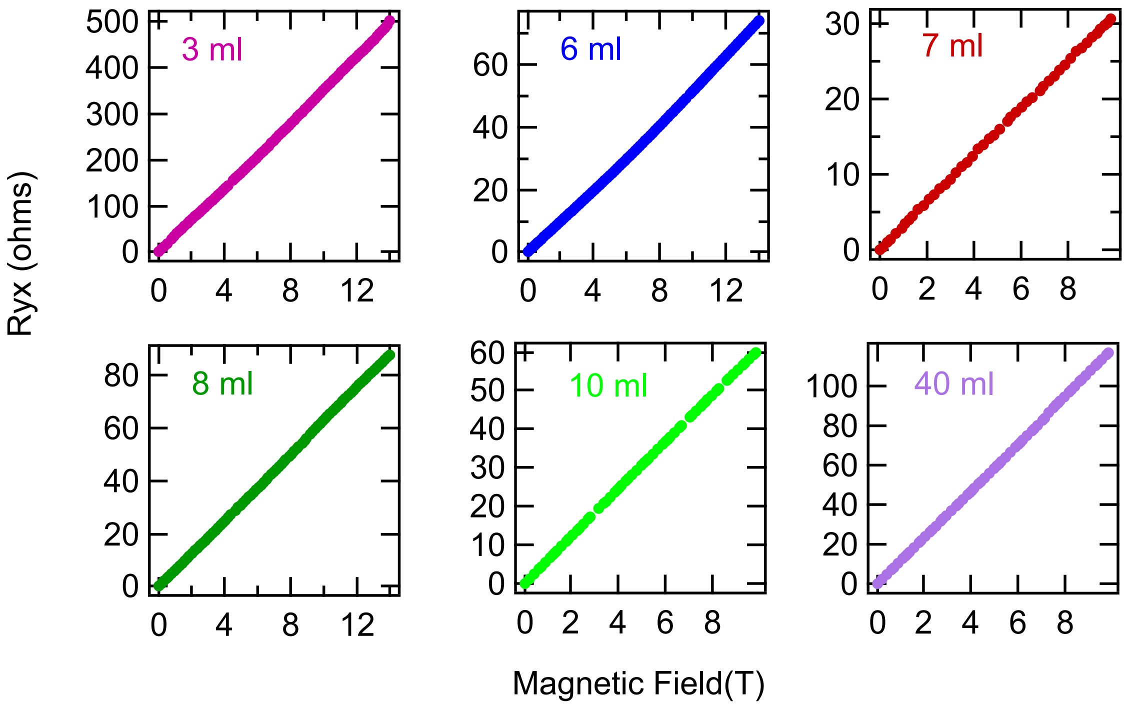

Supplementary Figure 3: **Hall measurements.**(Color online) Hall resistance for all the samples measured at 1.8 K. The linear dependence of Hall resistance suggests single type of carriers in the system.

non-monochromatic Mg K_α x-ray source having photon energy 1253.6 eV, the hemispherical analyzer operated at a constant pass energy of 20 eV and the pressure of analyzing chamber was maintained at $\approx$ 1x10^{-9} mbar throughout the measurement. All the spectra obtained were calibrated to a C 1s peak at 284.2 eV.[3] Core level XPS spectrum of LVO thin film is shown in the Supplementary Figure 4(a) below, where the obtained binding energy (BE) curve of V 2p core level depicting the two peaks corresponding to V $2p_{3/2}$ and V $2p_{1/2}$ at 516.0 and 523.6 eV BE respectively. As we have used the non-monochromatic Mg K_α x-ray source, this always gives Mg $K_{\alpha 3,4}$ signal in XPS spectrum which needs to be subtracted from raw data. The spectrum shown in the Supplementary Figure 4(a) is Mg $K_{\alpha 3,4}$ signal subtracted spectrum from the raw data of V 2p core level. There are several reports available in the literature for XPS peak position of the V^{3+} species ranging from 515.15 eV to 516.3 eV [4–7] and our result fall well within the reported range. We have further analysed V $2p_{3/2}$ core level spectrum by fitting it with the convoluted function. The shape of peak is defined by a Lorentzian function convoluted by a Gaussian function with full width at half maximum (FWHM) representing the lifetime and resolution broadening respectively.

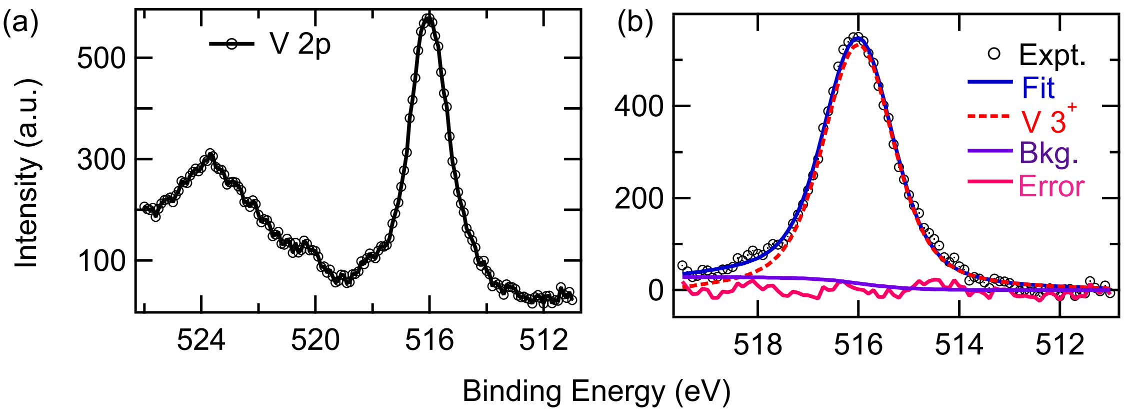

Supplementary Figure 4: **XPS measurements.**(Color online)(a) Core level X-ray photoemission spectrum of V 2p collected with Mg K_α x-ray source having 1253.6 eV photon energy (b) Fitting of V $2p_{3/2}$ peak using the convoluted function (Lorentzian function convoluted with Gaussian function).

In the Supplementary Figure 4(b), fitting of the V $2p_{3/2}$ peak is shown, where black open circles is the experimental data, blue solid line is the fitted spectrum, red dashed line is V^{3+} contribution in the spectrum, magenta solid line showing the error in the fitting and the violet solid line is depicting the Shirley background used for the background subtraction. We have deliberately omitted the V $2p_{1/2}$ part while fitting, since V $2p_{1/2}$ peak overlaps with tail part of the O 1s peak and that makes the fitting of whole V 2p spectrum more complicated. As shown in the Supplementary Figure 4(b), V $2p_{3/2}$ peak is fitted well with a single peak and this confirms the presence of the single valence state of V^{3+}.

In conclusion, XPS measurement and the fitting of V $2p_{3/2}$ core level peak confirm that V is present in single 3+ valence state.

---

[1] Trushin M., Vyborny K., Moraczewski P., Kovalev A.A., Schliemann J. and Jungwirth T., Anisotropic magnetoresistance of spin-orbit coupled carriers scattered from polarized magnetic impurities, Phys. Rev. B, **80**, 134405(1-14) (2009).

[2] Taskin A.A., Legg H.F., Yang F., Sasaki S., Kanai Y., Matsumoto K., Rosch A. and Ando Y., Planar Hall effect from the surface of topological insulators, Nat. Commun., **8**, 1340(1-7) (2017).

[3] Cardona1978 M. and Ley L., Eds. Photoemission in Solids I: General Principles (Springer-Verlag, Berlin) (1978).

[4] Mendialdua J., Casanova R. and Barbaux Y., XPS studies of V_2O_5, V_6O_{13}, VO_2 and V_2O_3, J. Electron Spectrosc. Relat. Phenom., **71**, 249-261 (1995).

[5] Sawatzky G.A. and Post D., X-ray photoelectron and Auger spectroscopy study of some vanadium oxides, Phys. Rev. B, **20**, 1546-1555 (1979).

[6] Maiti and Sarma D.D., Spectroscopic investigations of the electronic structure and metal-insulator transitions in a Mott-Hubbard system $La_{1-x}Ca_xVO_3$, Phys. Rev. B, **61(4)**, 2525-2534(2000).

[7] Hryha E., Rutqyist E. and Nyborg L., Stoichiometric vanadium oxides studied by XPS, Wiley Online Library, https://doi.org/10.1002/sia.3844, (2011).
